# Preparation of Effective NiCrPd-Decorated Carbon Nanofibers Derived from Polyvinylpyrrolidone as a Catalyst for H_2_ Generation from the Dehydrogenation of NaBH_4_

**DOI:** 10.3390/polym16202908

**Published:** 2024-10-15

**Authors:** Ayman Yousef

**Affiliations:** 1 Department of Chemical Engineering, College of Engineering and Computer Science, Jazan University, Jazan 45142, Saudi Arabia; aymanhassan@jazanu.edu.sa or aymanyosuef84@gmail.com; 2 Engineering and Technology Research Center, Jazan University, P.O. Box 114, Jazan 82817, Saudi Arabia

**Keywords:** electrospinning, nickel–chromium–palladium, carbon nanofiber, hydrogen, NaBH_4_

## Abstract

The catalytic dehydrogenation of NaBH_4_ for the generation of H_2_ has a lot of potential as a reliable and achievable approach to make H_2_, which could be used as a safe and cost-effective energy source in the near future. This work describes the production of unique trimetallic NiCrPd-decorated carbon nanofiber (NiCrPd-decorated CNF) catalysts using electrospinning. The catalysts demonstrated exceptional catalytic activity in generating H_2_ through NaBH_4_ dehydrogenation. The catalysts were characterized using SEM, XRD, TEM, and TEM-EDX analyses. NiCrPd-decorated CNF formulations have shown higher catalytic activity in the dehydrogenation of NaBH4 compared with NiCr-decorated CNFs. It is likely that the better catalytic performance is because the three metals in the NiCrPd-decorated CNF structure interact with each other. Furthermore, the NiCrPd-decorated CNFs catalyzed the dehydrogenation of NaBH_4_ with an activation energy (E_a_) of 26.55 KJ/mol. The kinetics studies showed that the reaction is first-order dependent on the dose of NiCrPd-decorated CNFs and zero-order dependent on the concentration of NaBH_4_.

## 1. Introduction

Hydrogen has gained significant interest as a sustainable energy carrier and a viable alternative to fossil fuels for various causes [1]. Hydrogen production from various sources has been a subject of considerable interest among scientists for a long time [2]. Hydrogen generation through the hydrolysis of chemically stored hydrogen is a straightforward and manageable process for generating hydrogen as required, with no concerns about storage and transportation [3]. The hydrogen storage material that has proven to be among the most valuable is sodium borohydride (NaBH_4_) [4]. Researchers have been highly interested in studying the hydrolysis of NaBH_4_ when a suitable catalyst is present [5,6,7,8]. NaBH_4_ offers numerous benefits, including a high gravimetric hydrogen content of 10.8 wt.%, a safe structure, and no toxic effects [6]. Borohydride hydrolysis is a promising method for generating hydrogen, which can be efficiently utilized by proton-exchange membrane fuel cells (PEMFCs) to provide power for various electronic devices such as vehicles, smartphones, and tablets [1]. Extensive studies have been conducted on the hydrolysis of NaBH_4_ for various applications, including on-board vehicular operation [9,10]. Studies have indicated that hydrogen storage and generation on-board vessels can be a feasible and reliable option. Until now, researchers have used a variety of catalysts to enhance hydrogen production through hydrolysis reactions [11,12]. Due to the scarcity and expense of noble metals like Pt, Pd, and Ru, scientists are increasingly interested in exploring catalysts made from more readily available transition metals like Co, Ni, and Cu [13,14,15,16]. These metals are cheaper and more abundant and effective for sodium borohydride (SBH) hydrolysis. Studies have found that bimetallic NPs exhibit higher catalytic activities in different chemical reactions compared with their counterparts [16,17,18]. This is believed to be due to the lattice geometry, strain, and electronic charge transfer influences [7]. Noble metals boost the catalytic activity of non-precious metals by creating synergistic interactions that enhance their electronic structure [19,20,21,22]. This improvement aids in the adsorption and activation of reactants like SBH and water. The addition of noble metals increases the number of active sites on the catalyst surface [21], facilitating easier interaction between reactant molecules and accelerating hydrogen production. By lowering the activation energy, noble metals make the reaction more energetically favorable, thereby enhancing efficiency at lower temperatures. Moreover, noble metals help stabilize the catalyst, preventing deactivation and maintaining performance over multiple cycles by reducing particle agglomeration. Furthermore, combining noble metals with non-noble transition metals enhances the catalytic activity and reduces the overall process cost [7,23,24]. Chunhui Yue et al. [19] demonstrated that the incorporation of palladium (Pd) into NiCo microfibers markedly improves their catalytic efficacy for hydrogen production from SBH. Pd-doped NiCo microfibers demonstrate a decrease in the E_a_ for the reaction, reducing it from 64.2 kJ/mol in the NiCo catalyst to 58.5 kJ/mol, thereby enhancing the kinetics of the reaction. Pd additionally improves the longevity of the catalyst, preserving more than 90% of its catalytic activity following 10 cycles of utilization. The integration of enhanced efficiency, diminished energy demands, and augmented durability positions Pd-doped NiCo microfibers as exceptionally effective catalysts for hydrogen production from SBH. Guo Z et al. [25] reported that the addition of noble metals increases the number of active sites on the catalyst surface. This effect was also observed in the 0.04 Rh_4_Ni/Al_2_O_3_ catalyst, where the Rh additive altered the chemical environment of Ni [26,27], leading to a higher proportion of metallic Ni in the bimetallic catalyst compared with the monometallic 4Ni/Al_2_O_3_ catalyst. Accordingly, the formed composite is also capable of the activating the cleavage of O-H bonds of water molecules [20,28]. Additionally, the geometric characteristics of these composites can be altered to increase the specific surface area and the assortment of active sites [21,29]. Recently, studies have introduced nano-trimetallic compounds as effective catalysts for the H_2_ production from SBH. For example, Wang et al. [30] synthesized copper–cobalt–nickel nanosheets through in situ reduction using SBH. Compared with the bimetallic copper–cobalt alloy, the catalytic activity toward H_2_ release from SBH was 1.3 times higher. Having two magnetic elements, Co and Ni, enhances the practicality of separating the solid catalyst from the liquid reaction solution. Patil et al. [31] developed a catalyst from iron–cobalt–copper oxides using the combustion synthesis process. The synthesized catalyst has shown promising results in catalyzing the release of H_2_ from SBH. The maximum rate of H_2_ generation is 1380 mL min^−1^ g^−1^, whereas the rates for the iron, cobalt, and copper oxides are 965, 226, and 126 mL min^−1^ g^−1^, respectively. Comparatively, the bimetallic iron–copper, copper–cobalt, and iron–cobalt oxides displayed values of 861, 784, and 756.3 mL min^−1^ g^−1^, respectively. From this perspective, the combined efforts of the three metals in the catalyst comprising the iron–cobalt–copper oxides achieved an impressive hydrogen generation rate (HGR). The catalyst consistently showed superior performance over eight cycles. Jiao et al. [32] prepared two different compositions of colloidal trimetallic NPs consisting of nickel, gold, and cobalt. Polyvinylpyrrolidone (PVP) shielded these NPs and synthesized them through an in situ reduction of metal ions using SBH. The catalytic performance of the prepared trimetallic formulations is compared to the bimetallic NiAu in the production of H_2_ via the dehydrogenation of SBH [33]. The Ni_50_Au_50_ catalyst exhibited a high hydrogen release rate of 800 mol-H_2_ per h per mol-M, while the Ni_50_Au_10_Co_40_ catalyst showed a slightly lower release rate of 790 mol-H_2_ per h per mol-M. The Ni_50_Au_10_Co_40_ compound exhibited lower activity compared with the bimetallic alloy. It is worth mentioning that Ni_50_Au_10_Co_40_ compounds have proven to be more cost-effective catalysts for H_2_ generation from SBH compared with those using Ni_50_Au_50_, especially when considering the percentage of gold. Catalytic metal NPs exhibit a tendency to aggregate due to their significant surface energy and magnetism. This tendency may lead to a reduction in the catalytic activity of the catalysts and a reduction in their lifespan. In this regard, the introduction of catalytic NPs into the matrix of supporting materials with higher specific surface areas (such as zeolite, nanocarbon-based materials, metal oxides, polymers, and so on) could improve the distribution of metal NPs without aggregation occurring. This could represent a suitable method for improving the properties of catalytic NPs [34,35,36,37,38,39,40,41,42,43,44]. Our team has conducted research on the use of CNFs as a catalytic support matrix for mono- and bimetallic NPs in the production of H_2_ from SBH and ammonia borane [45,46,47,48,49,50]. In this study, NiCrPd-supported CNFs were synthesized using the electrospinning approach and used as catalysts to produce H_2_ from the dehydrogenation of NaBH_4_. The catalytic performance of H_2_ generation from NaBH_4_ was evaluated by comparing the produced NiCrPd-supported CNFs with NiCr-supported CNFs under identical reaction conditions. The NiCrPd-supported CNF catalysts demonstrated notably superior catalytic performance in the dehydrogenation of NaBH_4_ compared with that by the NiCr-supported CNFs. Over a period of 10 cycles, the fabricated NiCrPd supported on CNFs demonstrated exceptional stability in producing H_2_ from NaBH_4_.

## 2. Experimental

### 2.1. Materials

Nickel(II) acetate tetrahydrate (NiAc, 99% purity; Aldrich Co., St. Louis, MO, USA) and chromium acetate dimer (CrAc, 98% purity; Aldrich Co., St. Louis, MO, USA) were used as metal precursors in the synthesis process. Polyvinylpyrrolidone (PVP, average molecular weight~1,300,000, 99% purity; Aldrich Co., St. Louis, MO, USA) was utilized as a polymer matrix. Sodium borohydride (NaBH_4_, 98% purity; Sigma-Aldrich, St. Louis, MO, USA) was used as a hydrogen source. Dimethylformamide (DMF, 99.8% purity; Sigma-Aldrich, St. Louis, MO, USA) and acetone (99.5% purity; Sigma-Aldrich, St. Louis, MO, USA) were utilized as solvents.

### 2.2. Preparation of the NiCrPd-Decorated CNF Catalyst

A PVP solution with a weight percentage of 15% was made by dissolving PVP powder in ethanol while stirring vigorously. A stock solution was formed by combining metal acetates (NiAc/CrAc) with a solution of PVP in a weight ratio of 1:3. A specific quantity of PdAc was introduced into 20 mL of the previous solution. The ultimate solutions were agitated at a temperature of 50 °C for a duration of 5 h and then cooled to room temperature in order to generate a sol–gel. The sol–gel was electrospun utilizing a 20 kV DC power source and a tip-to-collector distance of 18 cm. The fabricated nanofibers were gathered from a revolving cylinder that was covered with a polyethylene sheet. The electrospun nanofiber mats were dried for an extended period of time in a vacuum at a temperature of 50 °C. Ultimately, the mats underwent sintering in an argon environment for a duration of 5 h at a temperature of 900 °C, with a heating rate of 3 °C per min. The bimetallic material was likewise fabricated using identical processes.

### 2.3. Characterization

An examination of the morphology of the synthesized NFs was carried out with the help of a field-emission scanning electron microscope (SEM, Hitachi S-7400, Tokyo, Japan) and a JEOL JSM-5900 scanning electron microscope (JEOL Ltd., Tokyo, Japan). The characterization of the produced NFs was carried out using standard procedures. X-ray diffraction (XRD) using Cu Kα (λ = 1.54056 Å) radiation, which was supplied by Rigaku Co., Tokyo, Japan, was used to investigate the chemical makeup and crystalline structure of the NFs that were manufactured. For the purpose of acquiring high-resolution pictures, a transmission electron microscope (TEM) manufactured by JEOL Ltd. Tokyo, Japan with the model number JEM-2200FS and operating at a voltage of 200 kV and equipped with EDX was deployed [40,45,51].

### 2.4. Dehydrogenation of NaBH_4_

The SBH solution and catalyst were kept in a round-bottomed flask with two necks, one of which was stoppered while the other was connected to a gas burette. In order to control the temperature of the reaction, the apparatus was placed in an oil bath. A thermocouple was used to regulate the reaction’s temperature. The reactions were started by introducing 1.3 mmol of alkaline SBH and 100 mg of catalyst into a flask, which was then subjected to magnetic stirring at a speed of 1000 rpm at a temperature of 298 K. The gas volume was quantified using a gas burette by the method of water displacement. The amount of hydrogen produced was graphed as a function of time elapsed. The procedure was halted when there was no production of hydrogen gas. The same procedure was conducted without the inclusion of any catalytic material as a control experiment. All the generated catalysts underwent identical and stringent testing protocols. In order to conduct a more detailed analysis of the SBH hydrolysis kinetics, the experiment was conducted using various amounts of catalyst and SBH and with temperatures ranging from 298 to 313 K. An assessment was also conducted to determine the efficacy of recycling the suggested NFs. To evaluate the longevity of the catalyst, the procedure was iterated numerous times using the same set of catalytic NFs. The yield was calculated according to Equation (1).

Hydrogen yield (%) = generated hydrogen/theoretical hydrogen × 100(1)

Each cycle included the use of 1.3 mmol of SBH, 50 mg of catalyst, a temperature of 298 K, and a rotation speed of 1000 rpm.

## 3. Results and Discussion

### 3.1. Characterization of Hybrid Nanofiber Mats

Currently, researchers extensively use electrospinning technology to fabricate nanofibers from various metals, bimetals, trimetals, and their oxides owing to its affordability, efficiency, and ability to yield superior nanofibers. The chosen metal precursors are important because they can withstand polycondensation, which is needed to make an electrospinnable sol–gel with the right polymer. Precursors typically consist of metal alkoxides that undergo hydrolysis and polycondensation processes to form a cohesive network. Metal salts, such as chloride, nitrate, and acetates, can undergo hydrolysis and polycondensation, leading to the production of gel networks. Acetate has proven to be an excellent salt. Yousef et al. [52] outline the polycondensation reaction as follows:

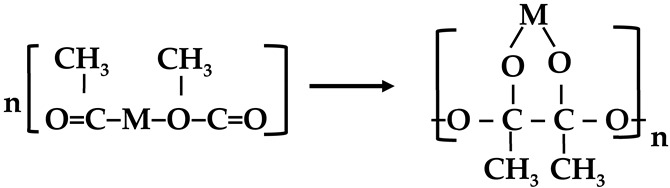



**Figure 1:**
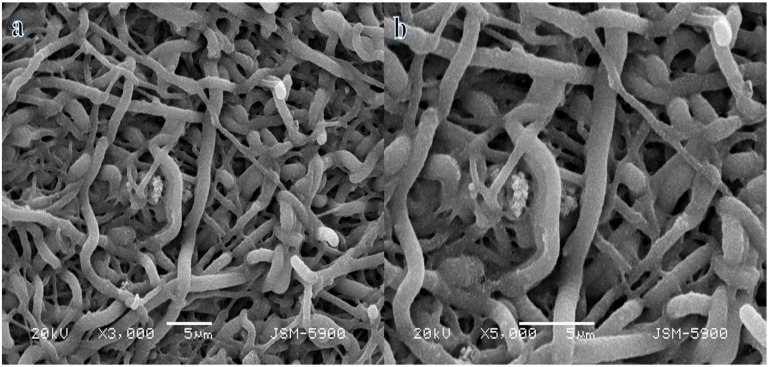
Low (**a**) and high (**b**) magnification SEM images of Ni_0.5_Cr_0.3_Pd_0.2_-decorated CNFs.

A metal is represented by the atom M. Thus, the metal precursors are utilized to generate extremely structured electrospun nanofibers. In addition, the nanofibrous morphology remains unaffected by the calcination process, as demonstrated in Figure 1. The SEM image of Ni_0_._5_Cr_0_._3_Pd_0_._2_-decorated CNFs (Figure 1a,b) for sintered nanofibers clearly illustrates this unique feature. The images show nanofibers with a rough texture as the nanoparticles began to grow and intertwine, resulting in the formation of a network of nanopores without beads and a well-defined nanofibrous structure. Despite calcination in an Ar atmosphere, the morphology of the calcined electrospun nanofibers remains intact.

**Figure 2:**
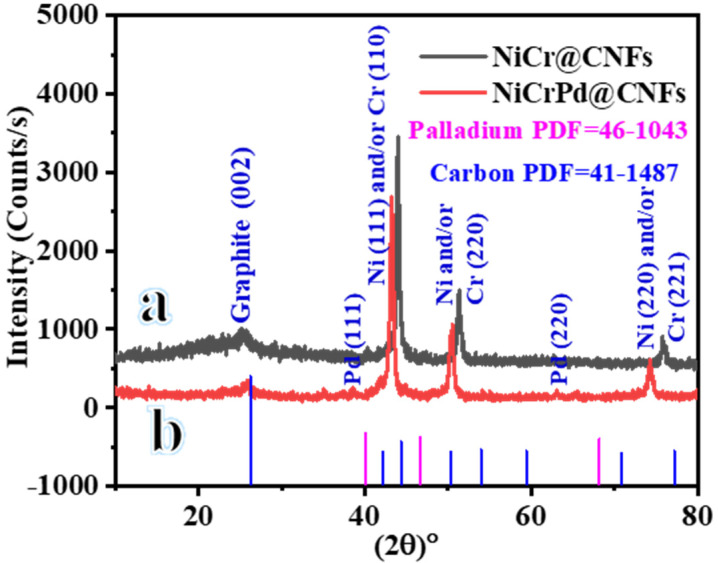
XRD of calcined NiCr (**a**) and NiCrPd (**b**) under vacuum in an Ar atmosphere at 900 °C.

Figure 2 displays the XRD patterns of the NiCr-decorated CNFs and NiCrPd-decorated CNFs. Ni and Cr are adjacent elements in the same period of the periodic table. At room temperature, they can form a solid solution up to a concentration of 30 wt.%. Beyond this threshold, a eutectic solution is produced. Because of the elevated melting points of Ni and Cr, namely 1455 and 1907 °C, none of the metals will evaporate during the carbonization process. The binary NiCr phase diagrams exhibit the significant solid solubility of Cr in Co and Ni. Furthermore, the NiCr alloys exhibit distinct crystal structures in comparison with the pure metals. The presence of Ni can be determined in the XRD pattern from the peaks at 2θ values of 44.5°, 51.8°, and 76.4°, which correspond to the crystal planes (111), (200), and (220) (JCDPS #04-0850). Similarly, the presence of Cr can be determined in the XRD pattern from the peaks at 2θ values of 44.4°, 64.6°, and 81.72°, which correspond to the crystal planes (110), (200), and (211), respectively (JCDPS #06-0694). The XRD patterns of the NFs produced show that the reflection peaks of Ni and Cr overlap, with little change in their angular locations. The inclusion of the comparatively bigger Cr atoms resulted in an expansion of the d-spacing in the Ni lattice. This caused a displacement of the Ni peaks toward smaller angles, as seen in Figure 2a. A similar pattern of peaks has also been shown by XRD of Ni_60_-Cr_40_, Cr, and Ni-20Cr [53,54,55]. The binary NiCr phase diagrams demonstrate significant Cr solid solubility in Ni. As a result, the peaks seen in the XRD pattern could be attributed to the presence of Ni and/or Cr. It is important to mention that pure Ni and Cr may coexist inside the same NPs, as confirmed by the TEM EDX investigation conducted below. The binary phase diagrams for the different alloying elements indicate that Pd exhibits high solubility in Ni [56]. The peaks in NiCr and NiPdCr correspond to reflections of a Ni solid solution with a face-centered cubic crystal structure. The addition of Cr and/or Pd caused a displacement of the peaks toward smaller angles in comparison with the conventional Ni spectra [56]. The inclusion of Pd in the NiCr alloy, mostly at the expense of Ni, caused the peaks to be further displacement toward smaller angles [56]. Figure 2b displays the findings of an XRD examination performed on NFs made of calcined PVP, CrAc, NiAc, and PdAc. In addition to the peaks that appeared in Figure 2a, the spectra show the presence of peaks at 2θ of 38.58° and 65.35°. These peaks are significantly shifted from cubic Pd (JCDPS# 41-1487, Sp.gr Fm3m (225)), which could be due to the formation of an alloy or solid solution between Ni-Cr-Pd. The crystalline size of NiCr@CNFs and NiCrPd@CNFs was determined used Scherrer equation [57], which was found to be 11.93 nm and 10.90 nm, respectively. In addition, there is a distinct peak at an angle of 2θ~26° in Figure 2a,b, indicating an experimental d spacing of 3.37 Å. This peak confirms the existence of carbon (d (002), PDF#41-1487). The findings indicate the formation of Ni, Cr, and Pd NPs-decorated CNFs.

**Figure 3:**
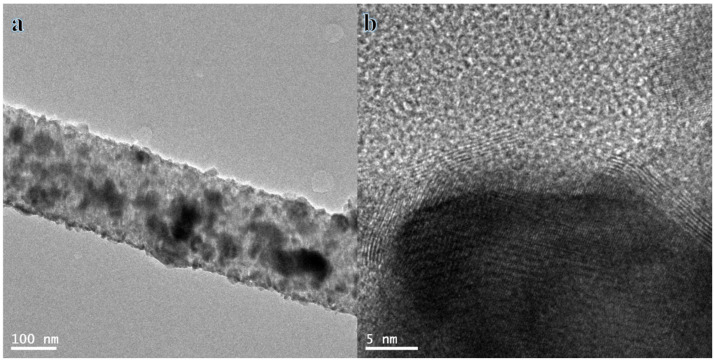
TEM (**a**) and HR TEM (**b**) images Ni_0.5_Cr_0.3_Pd_0.2_-decorated CNFs.

**Figure 4:**
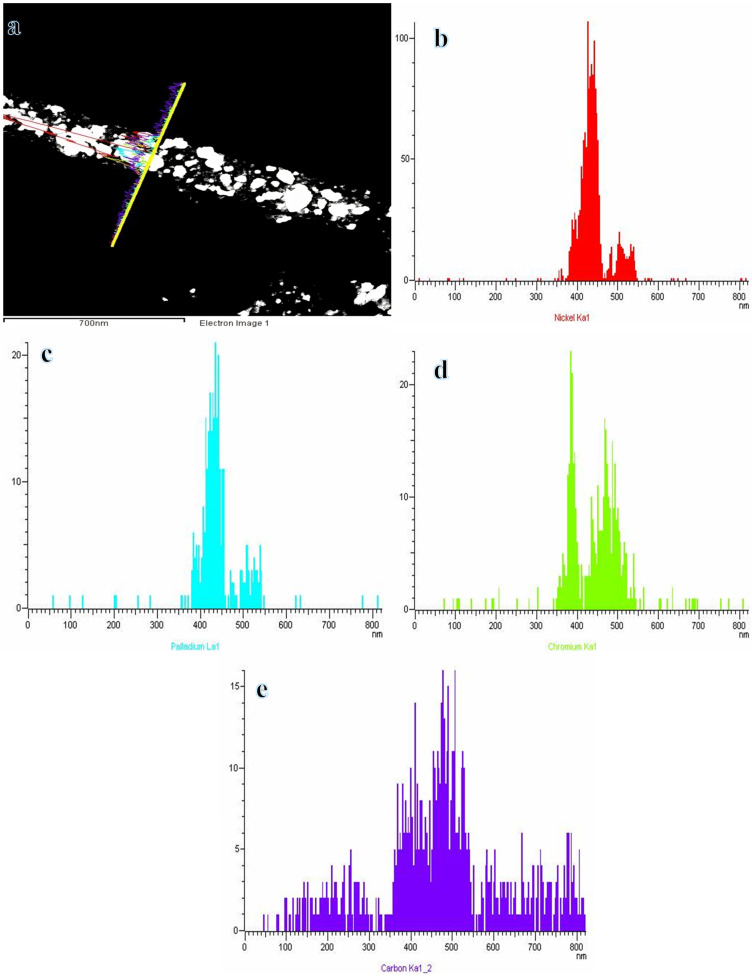
STEM image for NiCrPd-decorated CNFs (**a**) and the corresponding line TEM EDX analysis for Ni (**b**); Pd (**c**); Cr (**d**); and C (**e**).

The TEM images (Figure 3a) show that the NPs are distributed randomly among the surface of NFs. The HR-TEM image shown in Figure 3b indicates the presence of a thin layer of carbon with excellent crystallinity. This layer can enhance adsorption and electric conductivity, resulting in efficient electron transportation. It is evident from the observations in Figure 3b that the NPs are evenly distributed across the thin layer of CNFs. The distribution of Ni, Cr, and Pd throughout the formed NFs was investigated using linear analysis TEM EDX (Figure 4). Figure 4a demonstrates the distribution of Ni, Cr, and Pd along the selected line. Interestingly, the curves of Ni (Figure 4b), Cr (Figure 4c), and Pd (Figure 4d) show a distribution pattern for the metals. The different position of the prepared metals could be due to the alloying or forming the solid solution. Carbon forms the outermost element of the prepared NFs, as shown in Figure 4d. It can be inferred that CNFs have developed an outer shell around the metal NPs. It is quite simple for CNFs to adsorb NaBH_4_ and increase an electron transfer, which greatly facilitates the separation of H atoms.

### 3.2. Dehydrogenation of NaBH_4_

**Figure 5:**
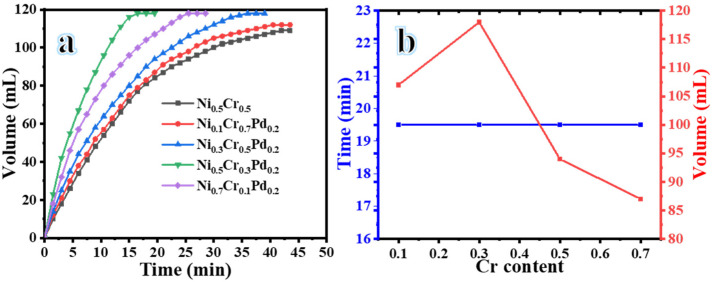
H_2_ evolution from NaHB_4_ using various formulations of NiCrPd-decorated CNFs (**a**) and the influence of an increase in Cr content on the dehydrogenation process (**b**).

NaBH_4_ is a chemical that contributes one of the two H atoms needed to form the final H_2_ molecule during the hydrolysis process. A proton derived from H_2_O provides an additional H atom [58]. The activation of a single O-H bond in the adsorbed H_2_O is the stage that determines the rate. The H_2_ bonding connection between a surface-coordinated BH_4_^−^ in the [BH_3_-H-H–OH] and a proton from H_2_O may facilitate the process of oxidative addition. This contact lowers the O-H bond’s electron density, which helps with the oxidative addition step. Furthermore, the superior conductivity of the CNF substrate enables the negative charge to move from BH_3_^−^ to H_2_ [59]. In the end, the catalyst’s surface will release the H_2_ via either reductive elimination or a coordinated σ-bond metathesis-like process involving a surface-coordinated BH4^−^ and a H atom from H_2_O, which is probably helped by a surface hydroxide ion (OH^−^) [60]. All NiCrPd-decorated CNF formulations were assessed as catalysts for the dehydrogenation of NaBH_4_. Figure 5a displays the time–volume plots of the H_2_ production through the dehydrogenation of NaBH_4_ process. The plots compare the different formulations of NiCrPd-decorated CNFs and NiCr-decorated CNFs, using the 1.3 mmol NaBH_4_, 0.1 g catalysts at 25 °C and 1000 rpm. The figure demonstrates that the NiCrPd-decorated CNFs exhibited superior catalytic activity for producing H_2_ compared with the NiCr-decorated CNFs. This can be attributed to the combined impact of the introduced Ni, Cr, and Pd. All NiCrPd-decorated CNFs exhibited superior catalytic activity for producing H_2_ from NaBH_4_, as depicted in Figure 5a, despite some variations in the H_2_ generation rates. Modifying the composition of the catalysts leads to the display of varying catalytic activities. The release rate of H_2_ was found to increase, while the duration time decreased, when the amount of Cr was increased from 0.1 to 0.3 (Figure 5b). Li et al. [61] demonstrated that introducing an appropriate amount of Cr to the Co-Cr-B/nitrogen-doped graphene catalysts caused a shift in the Fermi level’s position. The free energy and position of the valence band center changed because of this shift. This improved the H_2_-production performance from the dehydrogenation of NaBH4. The sample containing Ni_0.5_Cr_0.3_Pd_0.2_-decorated CNFs exhibits a faster release of stoichiometric H_2_ in comparison with other formulations, showcasing its superior catalytic activity. The determined generated H_2_ yields and H_2_ generation rate are demonstrated in Table 1. The high catalytic performance can be attributed to the synergistic interactions between Ni, Cr, and Pd, which improve electron transport. In addition, the presence of Ni, Cr, and Pd species alters the electronic structure of the mixture [39]. Pd could significantly improve the number of active sites in the prepared catalysts. Interpretation suggests that the combination of the Ni, Cr, and Pd is responsible for the significant increase in the H_2_ generation rate. This impressive performance can also be attributed to the CNFs. The CNFs possess excellent absorptive capacity and surface area and interact with a well-dispersed active catalyst, resulting in the formation of numerous active sites. It is widely recognized that electrospun CNFs possess a highly porous structure, allowing for easy interaction between reactants and active metals and facilitating H_2_ evolution. As a result, the nanofibrous morphology provides a significant increase in surface area and active sites for SBH dehydrogenation.

#### 3.2.1. Catalyst Amount

**Figure 6:**
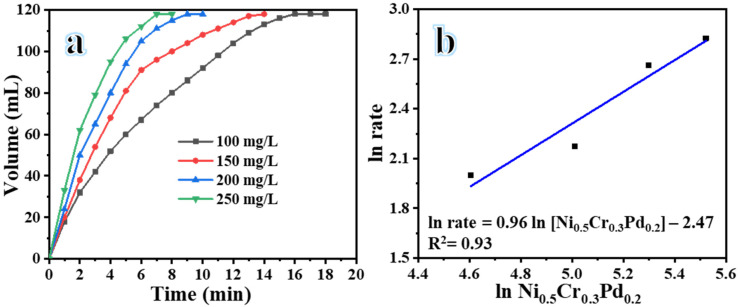
H_2_ evolution from NaHB_4_ catalyzed by different doses of Ni_0.5_Cr_0.3_Pd_0.2_-decorated CNFs (**a**), and the H_2_ generation rate versus the Ni_0.5_Cr_0.3_Pd_0.2_-decorated CNF dose (**b**).

Figure 6 illustrates the relationship between the H_2_ generation rate from the dehydrogenation of NaBH_4_ and the catalyst dosage. Doses of 100, 150, 200, and 250 mg/L of Ni_0.5_Cr_0.3_Pd_0.2_-decorated CNFs were used, while consistency was maintained for all the other conditions (i.e., SBH = 1.3 mmol at 25 °C and stirring speed of 1000 rpm). Figure 6a illustrates the impact of different doses of Ni_0.5_Cr_0.3_Pd_0.2_-decorated CNFs on the generated H_2_. As the catalyst dosage increased, the H_2_ production rate continued to rise (Table 2). As the quantity of the catalyst increases, the number of reactions that occur will also increase. Additionally, H_2_ generation may be less susceptible to the effects of catalyst deactivation [62]. This leads to an increase in the availability of surface active sites, which could be improve the dehydrogenation of NaBH_4_ [54,55,56]. The results generally show a direct relationship between the dehydrogenation of SBH and the Ni_0.5_Cr_0.3_Pd_0.2_-decorated CNFs catalyst. However, it is widely recognized that in any catalytic reaction, achieving high efficiency with a minimal catalyst dose is highly valued. The slope of the line in Figure 6b, which represents the relationship between the H_2_ generation rate and the catalyst dose, was 0.96 on a logarithmic plot. This demonstrated that the dehydrogenation of SBH follows first-order kinetics when using Ni_0.5_Cr_0.3_Pd_0.2_-decorated CNFs catalyst as the catalyst.

#### 3.2.2. NaBH_4_ Concentration

**Figure 7:**
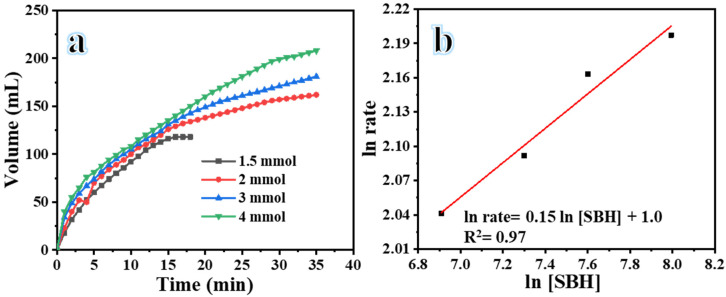
H_2_ evolution using different concentrations of NaHB_4_ (**a**), and the H_2_ generation rate versus the concentration of NaHB_4_ (**b**).

The study investigated the impact of varying the concentrations of NaBH_4_ (1.3–4 mmol), while keeping all other conditions consistent (0.1 g of Ni_0.5_Cr_0.3_Pd_0.2_-decorated CNFs, a temperature of 25 °C, and stirring at 1000 rpm), on its H_2_ evolution performance (Figure 7a). From the figure, it can be observed that the volume of H_2_ produced steadily increases with a rise in the concentration of SBH. On the other hand, the rate of H_2_ production does not show much variation when the concentration of NaBH_4_ is increased. As the concentration of NaBH_4_ increases, it results in higher water consumption and a significant increase in the production of NaBO_2_. This causes the solution to become viscous and reduces the contact area between the BH_4_^−^ and the Ni_0.5_Cr_0.3_Pd_0.2_-decorated CNFs catalyst, ultimately reducing the H_2_ release rate [61,63]. A logarithmic plot was developed to show the relationship between the H_2_ generation rate and the concentration of NaBH_4_, as depicted in Figure 7b. The slope of the line was determined to be 0.15. The results showed that the dehydrogenation of SBH using the Ni_0.5_Cr_0.3_Pd_0.2_-decorated CNFs catalyst followed a zero-order kinetics pattern that changed with the concentration of NaBH_4_.

#### 3.2.3. Reaction Temperature

**Figure 8:**
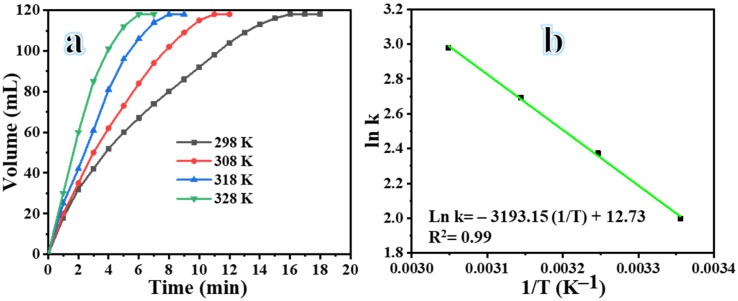
Effect of temperature on the dehydrogenation of NaBH_4_ (**a**) and logarithmic plot of the ln rate versus 1/T (**b**).

The reaction temperature greatly affects the catalytic hydrolysis of NaBH_4_ to produce H_2_ [64,65,66]. Figure 8a illustrates the significant increase in hydrogen generation as the temperature rose from 298 K to 328 K. All other factors remained unchanged, including the dose of the Ni_0.5_Cr_0.3_Pd_0.2_-decorated CNFs catalyst (0.1 g), the concentration of NaBH_4_ (1.3 mmol), and the stirring at 1000 rpm. According to the findings, the hydrogen release rate is positively correlated with the temperature of the NaBH_4_ solution. This is because as the temperature rises, the catalyst-to-NaBH_4_ solution contact area improves, leading to more rapid dissolution of the NaBO_2_ and, in turn, faster H_2_ release from the catalyst surface as well as a reduction in the deactivation of the catalyst [61,67]. The Ni_0.5_Cr_0.3_Pd_0.2_-decorated CNF catalyst exhibited initial activity at 298 K, with a high gas release rate of 7.38 mL min^−1^ (118 mL in 16 min). The catalyst’s substantial number of active sites allows for a greater rate of H_2_ production. This is due to the catalyst’s ability to efficiently activate the NaBH_4_ reactant and water molecules [31]. The H_2_ production rate showed a significant rise to 10.72 mL min^−1^ when the temperature was raised to 303 K, and the process reached equilibrium in a remarkably short time of 11 min. Through the application of heat, the interaction between the reactant, water, and the catalyst was enhanced, resulting in an accelerated reaction rate [31]. The H_2_ production rate showed an increase from 14.75 mL min^−1^ (118 mL in 8 min) to 19.66 mL min^−1^ (118 mL in 6 min) as the temperature was raised from 318 to 328 K. Based on the findings, it was observed that the H_2_ production rate demonstrated a direct correlation with the rise in temperature, suggesting that the process followed first-order kinetics. Figure 8b uses the Arrhenius equation to calculate the activation energy (E_a_), which is based on the initial rate of H_2_ production. The Arrhenius equation [64,65,66] indicates that the H_2_ production rate is related to the temperature and the activation energy (E_a_). The reaction seemed to exhibit first-order kinetics to the reaction temperature, as evidenced by the linear trend observed in Figure 8b. The Ea for this reaction was determined to be 26.55 kJ mol^−1^. The small E_a_ of the Ni_0.5_Cr_0.3_Pd_0.2_-decorated CNF catalyst allowed it to produce hydrogen quickly. The exceptional performance of the current Ni_0.5_Cr_0.3_Pd_0.2_-decorated CNF catalyst in hydrogen production is demonstrated through a thorough comparison with previous studies. Table 3 presents the catalyst’s remarkable catalytic efficacy in generating H_2_ from various precursors using trimetallic compounds.

#### 3.2.4. Recyclability Study

**Figure 9:**
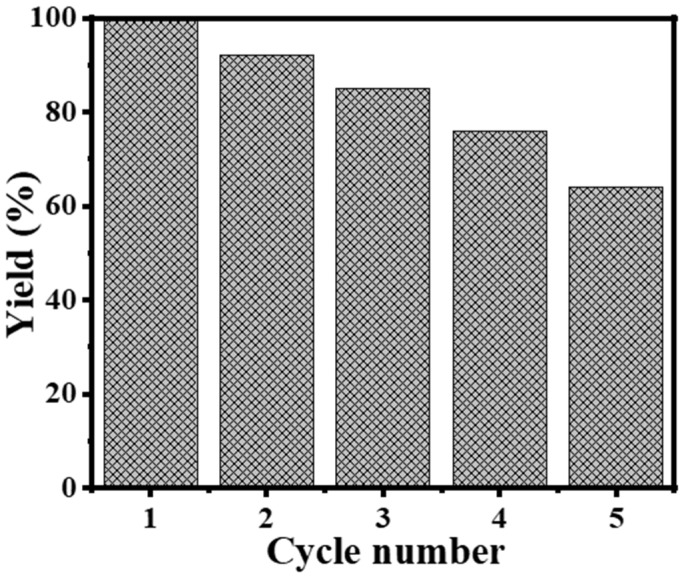
The recyclability of the Ni_0.5_Cr_0.3_Pd_0.2_-decorated CNF catalyst.

The results of the study on the recyclability of Ni_0.5_Cr_0.3_Pd_0.2_-decorated CNF catalyst can be seen in Figure 9. At 298 K, a reaction with the 0.05 g Ni_0.5_Cr_0.3_Pd_0.2_-decorated CNFs catalyst, 1 mmol NaBH_4_, and stirring at 1000 rpm released all of the H_2_. In the following cycles, 1.3 mmol NaBH_4_ was introduced to the reaction without the need for washing or supplying of the catalyst. The first three cycles demonstrated a consistent level of catalytic efficiency. During the experiment, the catalytic performance showed a gradual reduction, starting at 85% in the third cycle and reaching 64% by the fifth cycle. The FeCuCo catalyst demonstrated a retention of 80% of its initial catalytic performance after eight cycles [31]. The AC@Pt-Ru-Ni NPs exhibited a retention of 75% of its initial activity after undergoing three cycles [71]. The CoBMo/Cu catalyst demonstrated robust performance across five cycles, maintaining 98% of its initial activity [73]. The activated carbon@Pt-Ru-Ni NPs maintained 75% of their initial activity after three cycles [71]. This could be due to the rise in solution viscosity, which leads to a decline in the number of active sites or the blockage of pores due to the deposition of NaBO_2_ that cannot be removed by washing the catalyst, and reduced catalytic activity [74,75,76,77,78,79]. The findings indicate that the Ni_0.5_Cr_0.3_Pd_0.2_-decorated CNF catalyst used in the experiment demonstrated durability through five cycles of NaBH_4_ dehydrogenation without requiring any additional makeup or cleaning under experimental conditions. This suggests that it could be a promising material for catalyzing H_2_ production from the dehydrogenation of NaBH_4_.

## 4. Conclusions

The electrospinning technique was utilized to fabricate NiCrPd-decorated CNF catalysts. Catalytic activity was shown to be greatest in a sample consisting of Ni_0.5_Cr_0.3_Pd_0.2_-decorated CNFs when compared with other formulations. The synthesized Ni_0.5_Cr_0.3_Pd_0.2_-decorated CNFs showed a higher performance for H_2_ generation (1008.2 mol-H_2_ per mol-M) than the bimetallic Ni_0.5_Cr_0.5_-decorated CNFs (353.3 mol H_2_ per mol-M). Additionally, it is noteworthy that the catalyst exhibited a low activation energy of 26.55 kJ/mol, which is a remarkable value when compared with that of other tri-catalytic metals using various H_2_ storage materials that have been reported. This could be due to the synergistic interactions between Ni, Cr, and Pd, which improve electron transport and significantly enhance the number of active sites, ultimately leading to an increase in the total catalytic efficiency. In the process of recycling, it is noteworthy that the catalyst maintained decent activity for up to five recycling cycles without requiring any washing or makeup catalyst. This demonstrates that the catalyst is both effective and durable.

## Figures and Tables

**Table 1:** The H_2_ evolution rate from the dehydrogenation of NaHB_4_ using various formulations of NiCrPd-decorated CNFs.

Catalyst	Volume (mL)	Time (min)	Yield (%)	Rate (mL H_2_/min)	Rate (mol H_2_/h. mol Metal)
NiCr	109	46	90.8	2.51	353.3
Ni_0.7_Cr_0.1_Pd_0.2_	118	25	98.3	4.83	652.4
Ni_0.5_Cr_0.3_Pd_0.2_	118	16	98.3	7.15	1008.2
Ni_0.3_Cr_0.5_Pd_0.2_	118	36	98.3	3.27	462.1
Ni_0.1_Cr_0.7_Pd_0.2_	112	40	93.3	2.74	375.9

**Table 2:** H_2_ production rate from NaHB_4_ catalyzed by different doses of Ni_0.5_Cr_0.3_Pd_0.2_-decorated CNFs.

Catalyst Loading (g)	Volume (mL)	Time (min)	Yield (%)	Rate (mL H_2_/min)
0.1	118	16	98.3	7.38
0.15	118	14	98.3	8.8
0.2	118	11	98.3	14.3
0.25	118	8	98.3	16.86

**Table 3:** The comparison of the E_a_ of various tri-catalytic metals in H_2_ generation using various H_2_ storage materials.

Catalyst	E_a_ (KJ/mol)	Ref.
(Ni_5_Pt_5_)_1_-(CeO_x_)_0.3_/NGH	38.66	[68]
Ni_0.25_Fe_0.25_Pd_0.5_/UiO-66	43.5	[69]
Ni_45_Au_45_Co_10_	18.8	[32]
PdRuNi@GO	55.47	[70]
AC@Pt-Ru-Ni	24.29	[71]
Cu_0.04_Co_0.864_Ni_0.096_	40	[30]
Ru-capped/FeCo	42.9	[72]
Ni_0.5_Cr_0.3_Pd_0.2_	26.55	This study

## Data Availability

The original contributions presented in the study are included in the article, further inquiries can be directed to the corresponding author.
